# Mannan binding lectin triggers mobilization of hematopoietic stem cells

**DOI:** 10.18632/oncotarget.20705

**Published:** 2017-09-08

**Authors:** Mateusz Adamiak, Ahmed Abdel-Latif, Mariusz Z. Ratajczak

**Affiliations:** Mariusz Z. Ratajczak: Stem Cell Institute at James Graham Brown Cancer Center, University of Louisville, KY, USA and Department of Regenerative Medicine Warsaw Medical University, Warsaw, Poland

**Keywords:** complement cascade, mannan binding lectin pathway, hematopoietic stem cells, stem cell mobilization

Mounting evidence has accumulated that mobilization of hematopoietic stem progenitor cells (HSPCs) from the bone marrow (BM) into peripheral blood (PB) is a part of the developmentally ancient inborn innate immunity response to i) systemic or local infection, ii) stress related to tissue organ injury or iii) hypoxia [1-3]. In steady state conditions low numbers of HSPCs always circulate in PB and undergo a circadian rhythm in their circulation, with the peak occurring early in the morning and the nadir at night in a physiological process, a phenomenon that is also regulated by innate immunity [3]. All elements of innate immune including complement cascade (ComC), naturally occurring inborn IgM antibodies (NAbs), and Gr-1 + leucocytes [1-3] orchestrate the egress of HSPCs.

In clinical settings, the cytokine granulocyte colony stimulating factor (G-CSF) and the small molecular CXCR4 antagonist AMD3100, also known as Plerixafor may induce forced egress of HSPCs into PB and increase their number in PB up to 100 fold [1]. These cells mobilized by pharmacological means are than harvested from PB by leucopheresis as a source of HSPCs for hematopoietic transplants. Unfortunately, in autologous transplant settings ∼10% of normal patients and ∼25% of patients after chemotherapy do not respond efficiently to currently recommended mobilization protocols and are deemed poor mobilizers [1]. Therefore, it is important to better understand from a mechanistic point of view the mobilization process and to develop more efficient mobilization protocols in order to harvest the required number of HSPCs for successful transplantation.

The crucial role in this process plays activation of ComC that as it is well known may be activated by three pathways: i) the classical, ii) the alternative, and iii) the mannan-binding lectin pathway. When we initially discovered a requirement for ComC activation in HSPC mobilization [3,4] we assumed that the classical activation pathway of ComC would play a pivotal role in the entire mobilization process. Nevertheless, somehow to our surprise, mice with inherited mutations in components of classical pathway did not show impairment in the mobilization of HSPCs [3]. Therefore, we turned our attention to the somewhat understudied mannan binding lectin (MBL) pathway of ComC activation and our most recent work with MBL deficient mice (Mbl^-/-^) revealed that in fact this pathway and not the classical pathway triggers a ComC-mediated mobilization process [5].

MBL belongs to a family of circulating in biological fluids soluble pattern-recognition receptors (PRRs) that recognize two classes of molecules, namely i) pathogen-associated molecular pattern molecules (PAMPs), which are expressed by microbial pathogens, and ii) damage-associated molecular patterns molecules (DAMPs), which are associated with cell components and are released during cell activation, cell damage, or cell death.

It is known that MBL is a major PRR of the innate immune system and besides binding to a wide range of pathogens also recognizes phospholipids modified by free radicals (ROS) as well as several DAMPs released from activated cells, such as high-mobility group box 1 (HMGB1), extracellular ATP, DNA, and hyaluronian fragments [5]. Once bound to ligands, MBL recruits MBL-associated serine proteases (MASP-1 and -2) and initiates first enzymatic activation of the ComC by targeting C3 component, leading finally after several steps to the generation of C5 cleavage fragments C5a and iC5b that are crucial to execute egress of HSPCs from BM [4].

**Figure 1:**
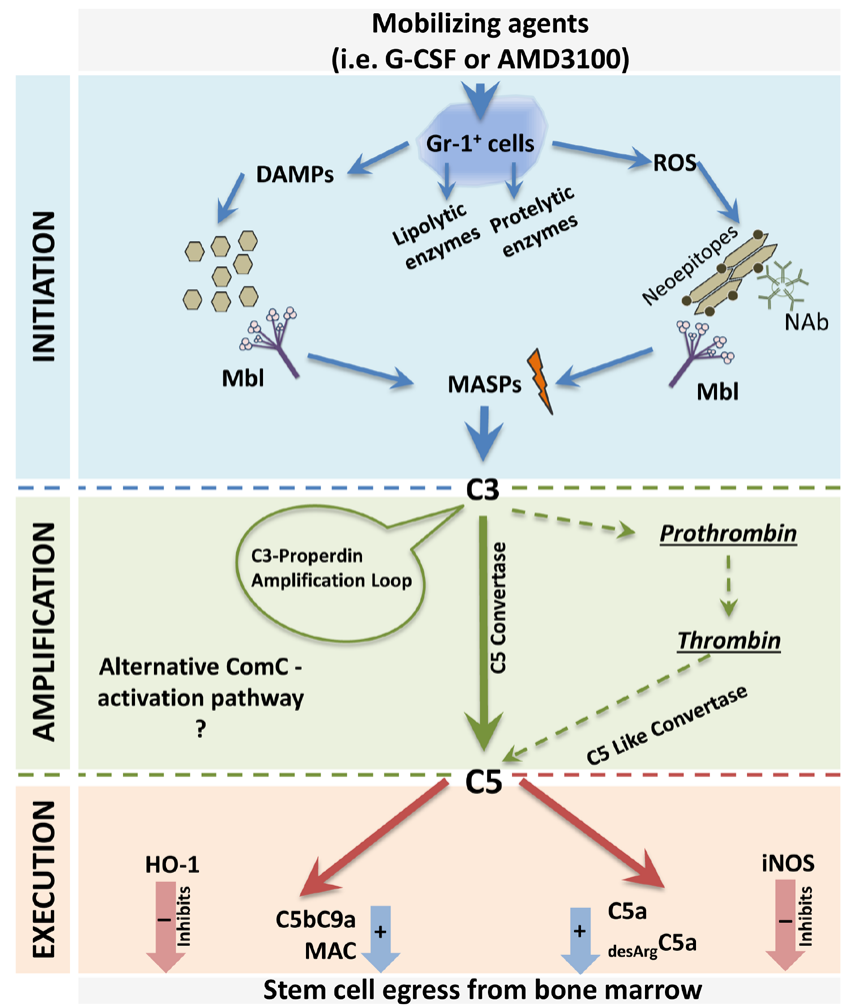
Proposed MBL-induced three-step model for triggering the mobilization of HSPCs All the phases of mobilization process are depicted here. Step I (initiation phase). Activation of Gr-1^+^ neutrophils and monocytes by a mobilizing agent (e.g., G-CSF or AMD3100) induces the release of proteolytic and lipolytic enzymes and the secretion of ROS and DAMPs by these cells. In the BM microenvironment while secreted enzymes attenuate the retention of HSPCs in BM niches, and ROS expose neoepitopes. Neoepitope–IgM complexes as well as DAMPs are recognized by MBL, which activates the ComC and CoaC in a MASP-dependent manner. Step II (amplification phase). Convertases (classical C5 and C5-like) generated in this step cleave C5 to release cleavage fragments crucial in egress of HSPCs from BM. This step is also modulated by a C3 auto-amplification loop, with a possible contribution from the alternative ComC-activation pathway. Step III (execution phase). In this step, C5 cleavage fragments C5a and _desArg_C5a promote the release of HSPCs from BM, and this process is negatively regulated by HO-1 and iNOS.

Figure 1 depicts step by step the pivotal involvement of innate immunity in the mobilization of HSPCs. For simplicity reasons we divided this process into i) initiation, ii) amplification and iii) execution phase. The first initiation step starts with the activation of Gr-1^+^ granulocytes and monocytes by mobilizing agents (e.g., G-CSF) and leads to the release of proteolytic and lipolytic enzymes by these cells that disrupt retention/adhesion interaction between HSPCs and BM stem cell niches. The enzymatically affected retention proteins involve ligand-receptor SDF-1–CXCR4 and VCAM-1–VLA-4 interactions [3-6]. In parallel activated Gr-1^+^ granulocytes secrete reactive oxygen species (ROS) that expose auto-antigens known as “neoepitopes” in the BM microenvironment, which bind above mentioned NAbs, mainly of the IgM class [3,5]. In addition, Gr-1^+^ cells also release soluble DAMPs including HMGB1, extracellular ATP, DNA, and hyaluronan fragments. Both modified by ROS phospholipid neoepitope-NAb complexes as well as released DAMPs are recognized by MBL that via MASPs activates the ComC to generate C5 convertase to cleave C5 and C5 cleavage fragments anaphylatoxins C5a and _desArg_C5a. Both these molecules regulate the execution phase of HSPCs mobilization (Figure 1) and facilitate egress of cells from BM by permeabilizing the endothelial barrier in BM sinusoids [4].

The MBL induced ComC activation is additionally supported as we propose during the amplification phase (Figure 1) by co-activation of the alternative pathway of ComC [3,5]. MBL-MASPs also activated coagulation cascade that potentiates mobilization process [3,7]. On the other hand mobilization has to be negatively regulated and both heme oxygenase-1 (HO-1) and inducible nitric oxide synthetase (iNOS) both play an important inhibitory role [3,8].

In conclusion, our most recent data on a crucial role of MBL in inducing the mobilization process also have potential important clinical implications. As mentioned above ∼10% of normal patients are poor mobilizers of HSPCs and since MBL-directed ComC-activation pathway plays a crucial role in triggering the mobilization of HSPCs, one can ask if MBL-deficiency may explain the poor mobilization status of some patients. In fact, human MBL deficiency, the most common form of complement deficiency, is seen in 5–10% of the population [1], and correlates with the percentage of poor mobilizers. Thus, MBL could potentially serve as a good biomarker to identify poor mobilizers. Our observations are also highly relevant for other processes in which an increase in stem cell trafficking occurs, such as for example in stress related to infection, tissue/organ injury, or strenuous exercise - as all these processes activate ComC.
